# Garden on the Go: A Feasibility Study of a Gardening Program to Support Mental Health and Resilience in Youth with Adverse Childhood Experiences

**DOI:** 10.3390/children12111444

**Published:** 2025-10-24

**Authors:** Glenda E. Hux, Sydney Rice, Amy Wagenfeld, Sarah A. Schoen

**Affiliations:** 1 Department of Communication Disorders and Occupational Therapy, College of Education and Health Professions, University of Arkansas, Fayetteville, AR 72701, USA; sbrice@uark.edu; 2 Department of Occupational Therapy, College of Health Professions, University of Arkansas for Medical Sciences, Fayeteville, AR 72701, USA; 3 Department of Landscape Architecture, College of Built Environment, University of Washington, Seattle, WA 98105, USA; awagenfe@uw.edu; 4 Department of Occupational Therapy, Rocky Mountain University of Health Professions, Provo, UT 84606, USA; sarah.schoen@rm.edu

**Keywords:** occupational therapy, gardening, adverse childhood experiences, sensory processing, education, mental health and well-being

## Abstract

**Background/Objectives:** The benefits of nature-based interventions to support well-being and mental health are increasingly well-documented in the literature; however, study of an occupational therapy gardening program for adolescents with exposure to adverse childhood experiences (ACEs) is limited. **Methods:** This study evaluates the feasibility of a novel school gardening program for youth with a history of ACEs including the following: (1) recruitment; (2) data collection procedures and outcome measures; (3) acceptability and suitability of the intervention; and (4) evaluation of the response to a gardening intervention as measured by a visual analog scale of emotional state, a heartbeat counting task designed to capture changes in interoceptive awareness, and qualitative data from the teacher and researchers. This feasibility study was designed as an 8- to 10-week program (10 sessions minimum) to accommodate the school’s academic curriculum and support the participants’ academic progression. Three adolescents were recruited, ages 12–17, two of whom completed a shorter version of the program and one who dropped out. **Results:** Results indicated the gardening intervention recruitment and data collection procedures were feasible. Intervention was acceptable to participants. Outcome measures that produce both quantitative and qualitative changes are needed. Interoceptive measures show promise but require further refinement. Response to intervention seemed to be influenced by the participant’s psychosocial history but suggests possible changes in prosocial behavior. External factors such as absenteeism influenced aspects of participation, including frequency and duration of intervention. **Conclusions:** These findings suggest gardening interventions in occupational therapy are feasible and suitable for adolescents with a history of adversity. Potential exists for enhanced social connectedness, which supports mental health and well-being. Suggestions are offered for implementation and outcome measurements appropriate for this population.

## 1. Introduction

Adolescence is characterized by neurological reorganization with profound impact on emotional regulation, motivation, socialization, and sensory processing [1]. Adverse childhood experiences (ACEs) such as the presence of divorce, depression, substance use, physical threats, household insecurity, sexual abuse, or incarcerated parents may exacerbate the existing challenges these youth encounter. In fact, these adolescents are at risk for impaired processing of sensory signals [2] that contribute to functions related to regulation, cognition, and social emotional abilities. For example, youth who experience childhood adversity are more likely to report challenges such as difficulty focusing on specific tasks, controlling their impulses, and perceiving bodily signals [3,4,5]. ACEs may further disrupt the relational and physiological developmental cycle of attachment, influencing affect, cognition, and behavior, including increasing isolation and higher rates of suicide, substance use, and disembodiment [2,6]. Therefore, a need exists to provide feasible, acceptable, and effective interventions programs to support these at-risk adolescents.

### 1.1. Trauma, Interoceptive Awareness, and Social Participation in Adolescents

Interoceptive awareness, the body’s conscious awareness of internal body sensations like hunger, thirst, or arousal, is an integral part of the somatosensory system and is often impacted in adolescents exposed to stress. Notably, individuals with higher levels of trauma experience and exposure to acute stressors [7] are at greater risk of impaired physiological, psychological, emotional, and social processes linked to awareness of bodily signals that influence self-regulation and behavior [8]. These impaired processes may hinder relational rupture and repair [9] and the adolescent’s ability to form and maintain relationships and a safe therapeutic alliance with mental health and related professionals [10,11]. Thus, interoceptive abilities may provide a window into the interoceptive functions related to adolescent functions across biopsychosocial domains.

Additionally, interoceptive interventions may provide support to interoceptive functions that support these biopsychosocial domains. Kumar [12] demonstrated a statistically significant impact of interoceptive programs on academic self-regulation and behavioral outcomes in children with learning disabilities. Studies of interoception in clinical psychology suggest that bringing attention to internal bodily sensations may impact emotional processing [13]. Further, Lynch [11] and Schmitt & Schoen [14] suggest that an understanding of the physiological mechanisms of interoception by occupational therapists is an important consideration when designing effective intervention programs.

### 1.2. Nature’s Multidimensional Role in Supporting Biopsychosocial Development

The positive influence of nature, gardening, and therapeutic horticulture on human behavior and its connection to mental and emotional well-being is well-established in the recent literature [15,16]. Of primary importance is the dynamic, bidirectional influence between person and environment when engaging with nature [15,17]. Benefits include aspects of psychological, cognitive, physical, and emotional well-being, as well as social connectedness related to quality of life and a reduction in mental health symptoms of depression, stress, and anxiety [16]. Most notably, there are reports of improved mental resilience from participation in a weekly structured, therapeutic gardening program, especially when engaged for at least one hour a week [18]. Examples of specific components found in nature to produce therapeutic benefit includes universally designed environments, sensory-enriching experiences, and an intrinsic motivation to garden [19]. These benefits may occur with active engagement with nature-related activities, which can improve affective and cognitive processes [20] as well as sustained attention and learning [21]; these serve as protective factors to help individuals cope with adversity and build resilience [22]. Likewise, the multisensory elements of nature-related activities (i.e., bird sounds, fragrance, sunlight, soil, water, and colorful flowers) may support active engagement in the therapeutic process [23].

### 1.3. Meeting the Biopsychosocial Needs of Youth Exposed to Childhood Adversity

Disproportionately, students in alternative learning environments (ALEs) have experienced social, emotional, cognitive, and behavioral challenges resulting in unsuccessful engagement in academic tasks in traditional classrooms. These challenges include traumatic experiences, aggressive externalizing behaviors, lack of adequate attendance, and decreased academic success [24]. Programming for youth exposed to childhood adversity should provide opportunities for enriched sensory play and leisure environments to positively impact trusting alliances, resilience, and academic success [25]. Importantly, nature-based experiences have been demonstrated to enhance social emotional behaviors [26] and positive youth development [27].

Additionally, engagement in activities with shared goals [28] and social leisure activities during adolescence may be critical therapeutic features that potentially buffer the adverse effects of ACEs on mental health [29]. However, a current lack of interventional studies limits our understanding of the effects of nature-related interventions on interoception and emotional state. There is also a need to further explore the role of social and inclusive sensory-enriched nature environments, social leisure activities in nature, and sensory–motor recreation in supporting meaningful participation in youth experiencing adversity within educational and community settings. In preparation for a future study, this study was designed to explore the feasibility and acceptability of a structured, therapeutic gardening program for adolescents exposed to ACEs. Secondarily, this study sought to evaluate this gardening program with respect to potential modifications and improvements that may need to be made to maximize student outcomes.

### 1.4. A Structured, Therapeutic Occupational Therapy Gardening Program

An occupational therapy intervention was developed that uses gardening as the therapeutic medium as detailed in Section 2.8. Prior to the start of the intervention, the team discussed with the school’s personnel how to accommodate the research project within the structure of the school’s curriculum so as to not interfere with the student’s curricular progression. The research team and the school personnel agreed the program would take place during the first 8 weeks of the 16-week semester for a minimum of 10 sessions. The recommended setting was one with a large green space that has access to a water source and secures storage of tools. Workstations and mobile materials were needed as well as enhanced communication strategies when working with students, such as whiteboards, pictures, and other visual cues to support successful participation.

### 1.5. Aims of the Feasibility Study

Thus, this feasibility study was designed to achieve the following aims [30]:

1. Evaluate recruitment capabilities and resulting sample characteristics in an educational environment with a prevalence of ACEs.
2. Evaluate and refine data collection procedures and outcome measures using objective physiological (heartbeat counting task (HCT) for interoceptive accuracy (IAc)) and subjective behavioral outcome measures of emotional state (visual analog scale) in youth exposed to ACEs.
3. Evaluate acceptability and suitability of intervention and study procedures for a structured, therapeutic gardening program in an educational environment with youth exposed to ACEs.
4. Preliminary evaluation of participants’ response to a gardening intervention (limited efficacy testing) in youth exposed to ACEs.

## 2. Method

### 2.1. Research Design

This mixed-methods feasibility study follows the recommendations of Orsmond & Cohn [30] for developing and testing a novel intervention. The first step was to evaluate the acceptability and suitability of a structured, therapeutic gardening program in an educational environment. The study collected quantitative data to assess feasibility characteristics, measurement of physiology, as well as qualitative data from ethnographic observations by researchers and semi-structured interviews of teachers. The research team included the principal investigator (PI), an occupational therapist with over 20 years of experience; a third-year occupational therapy doctoral student researcher (co-PI), and an occupational therapist and subject matter expert who served as a consultant due to her extensive experience in nature-related interventions, health promotion, and therapeutic landscape design.

### 2.2. Recruitment Capabilities and Sample Characteristics Method

Recruitment efforts took place in a full-time alternative educational environment (ALE) in a suburban community in a rural south-central state. All potential participants met the following criteria for full-time placement in the ALE: (1) personal or family problems; (2) recurring absenteeism; and (3) ongoing, persistent lack of attaining proficiency levels in literacy or mathematics. A pre-determined goal was a sample with a minimum of three and a maximum of five participants due to the students’ need for close supervision. Feasibility was assessed by reporting the number of interested students, articulating eligibility criteria, and determining the suitability of the intervention. All recruitment procedures were conducted with prior authorization from the school’s educational leadership and were sanctioned by the IRB who approved this study.

### 2.3. Recruitment and Sample Results

Results of recruitment efforts identified interest and motivation from a convenience sample of 30 enrolled students. Twenty potential participants and families attended the back-to-school meeting and were informed about the study. In total, 9 of 20 (45%) full-time students expressed interest in participating in the study. Six of nine students signed consent and assent forms during the first two weeks of school. Therefore, we concluded that the eligibility criteria did not negatively impact enrollment and that the recruitment procedures and setting were feasible and relevant for the population.

The specific inclusion criteria were based on the criteria for admission to the ALE and was successfully applied as follows to those individuals/families who signed consent and assent: (1) no recorded disciplinary or safety concerns in the last academic year per record review or teacher report; (2) self-reported interest in gardening; (3) at least one challenge in participation in the student role (i.e., communication abilities, successful activity performance, positive classroom habits and/or productive peer interactions) identified by teacher; and (4) minimum line of sight supervision required with use of simple gardening tools per record review or teacher report.

Three (n = 3) students were selected who met these inclusion criteria and agreed to participate in the study. Participants’ ages ranged from 12 to 17 years. The research team ensured that educational counselors and/or community mental health supports were accessible to all participants as stipulated by their educational team. Sample characteristics determined that the intervention was relevant to the study participants. Additional participant characteristics appear in Table 1.

### 2.4. Data Collection Procedures and Outcome Measures Method

The data collection procedures and outcome measures were assessed based on the ability of measures to be completed, whether data were complete and usable, and whether the measures were appropriate for this population and relevant for the intervention. Procedures involved quantitative data collection, which included pulse oximeter and a heartbeat counting task as the physiological measures, as well as subjective participant reports of emotional state (developed by Champagne, [31] and published by Dowdy et al. [32] for adolescents with a history of ACEs). Qualitative data were collected through ethnographic observation by the two researchers (e.g., PI and OT student), first in the classroom before a gardening session and then secondly during a gardening session. The PI trained the OT student in all procedures. As the study did not focus on teachers as recipients of the intervention, teachers were not present during the gardening sessions. Semi-structured interviews were conducted by researchers with the participants’ homeroom teachers only, taking place the day after gardening sessions to ascertain students’ changes in classroom behaviors and student outcomes.

Data were collected at different stages of participation: (1) a within-session recording of heart rate physiology (e.g., pulse and heart rate counting) was collected before a gardening treatment session and then after participation in the session, (2) behavioral observations were collected in the classroom before a gardening session and then during gardening, (3) teacher feedback was obtained after each gardening session through 1:1 meeting with a member of the research team using open-ended questions about student performance, and (4) physiological data were collected across sessions in order to explore changes from the beginning to end of the intervention program.

#### 2.4.1. Data Collection Measures

##### Heart Rate

Two measures were used to collect heart data: the heartbeat counting task (HCT, [33]) and pulse rate oximeter. Data were collected on emotional state using a visual analog scale, and qualitative data were obtained from researchers’ observations and teacher interviews.

The HCT reflects the individual’s accuracy of self-reported heartbeats to actual heartbeats in beats per minute [33]. Previous research suggests that exposure to acute stressors (e.g., micro traumas) can impair one’s ability to perceive their own heartbeat accurately [7].

The HCT was the physiological measure of interoceptive awareness that was assessed for feasibility and accessibility/appropriateness to the population and the setting. Testing took place in a quiet classroom at baseline and in the gardening area during the sessions. The participants received instructions to stay as still as possible and to count only the heartbeats they felt without touching anywhere on their body to feel their pulse [34]. Verbal instructions were adapted from Desmedt et al. [34]; participants were signaled when to begin counting [34]. Participants were unaware of the time intervals for recording heartbeats. Intervals were randomized (25, 35, 45, or 55s) each session to prevent the chance of participants predicting the length of time for data acquisition. The interoceptive accuracy score is determined by how well the reported heartbeat score corresponds to the heartbeat count measured by the researcher [34]. Each interoceptive accuracy score was based on a comparison of the pulse oximeter measure compared to the reported number of heartbeats by the participant. The HCT score was calculated as the average score across intervention at both pre- and post- treatment sessions. HCT scores range from 0 to 1, with higher scores considered as having greater interoceptive accuracy.

The pulse oximeter measure was used to record the data of pulse rate collected by the researcher as an additional physiological measure. A researcher used a pulse oximeter to measure pulse rate in beats per minute (BPM). Repeated measurements of pulse and HCT were taken at the start and end of each session to capture in-session changes for each participant as well as across the intervention program. Average scores were compared across intervention to explore differences between pre- and post-intervention.

##### Emotional State

A visual analog scale (VAS) was used to assess emotional state within each session during the program. The participants used the VAS to rate their subjective emotional state on a scale of 0–10 as well as using red and green emoticons to symbolize the intensity and the zone of emotion chosen. Lower scores represented a more positive emotional state. Scores were calculated based on the average of pre- and post-session measurements. Participants completed a verbal description of their emotional state along with these ratings. The tool asked students to answer a Likert scale [35] question rating from zero to ten how they felt post-session and to write a word describing how they felt (e.g., happy, tired, sad, relaxed, annoyed or okay).

##### Qualitative Behavioral Data

Ethnographic observations and field notes were collected throughout the study by the researchers. Observations were conducted in the classroom as well as during gardening sessions. Additionally, researchers obtained feedback from the teacher about student performance following the gardening sessions. Indicators of in-session responses within affective, physiological, and/or social/spiritual domains were noted. Observations were focused on enhancing identification of behavioral outcomes following intervention activities including social interaction, task initiation and persistence, task participation, task engagement, and successful completion of tasks. To minimize bias, reflective memos were used to document the presence or absence of behaviors post-session. Any discrepancies in the data (i.e., terminology or presence of behavior) recorded by the principal investigator were discussed by the research team until consensus was reached. Teacher feedback was solicited after each session to capture student in-class changes following each treatment session.

### 2.5. Data Collection Procedures and Outcome Measures Results

Student participants were able to complete the HCT and the VAS with no missing data, except for when they were absent from a given session. These measures required approximately 2 min per participant to complete. Students needed some assistance from the researcher for encouragement and supervision while completing the measures. Although there was full participation in completing the measures, accuracy for the HCT was variable (as this was the first time this task was used with this population). Pulse oximeter readings were consistently recorded within the session and across sessions by the researcher.

Participants also successfully completed VAS measurement of emotional state each session. However, additional directions were needed to ensure responses were relevant and accurate as follows: zero (0), represented by a green happy face, was the equivalent of the best you have ever felt, and 10, represented by a red (frowny) face, was the equivalent of the worst you have ever felt. While there seemed to be little variety across and within sessions, emoticons and verbal reflections helped to clarify the nature of participant responses. For example, one participant described a fight he had with his girlfriend that influenced his emotional state during one of the gardening sessions. Interestingly, reports of fatigue also elicited changes in value and emotional state reported.

Participant data were successfully obtained in collaboration with the classroom teacher, which included teacher reports, and through classroom observations by the researchers following each intervention session. Teacher meetings post-session provided essential feedback on participant gains/changes related to concerns identified at the start of the study. Teacher feedback was provided in the form of informal interviews, while researcher observations were in the form of detailed written records of observation in the classroom before participating in a gardening session and then during the gardening session.

Overall, the measures were feasible, suitable, and appropriate for the study. Additional training in heart rate monitoring is suggested to increase accuracy. Practice during a no-intervention phase and teach-back method would also increase accuracy of the VAS emotional scale. In general, use of strategies that ensure participants understand the measures provided would be helpful in improving adherence to the data collection procedures [36].

Additionally, a more systematic approach to collecting qualitative data from the researcher, teacher, and participant is recommended. Specifically, a short feedback form or checklist might better capture a wider range of behavioral changes from researcher and teacher. Recommended modifications include use of a verbal self-report or checklist to provide more consistent and formalized feedback from the participant.

### 2.6. Intervention and Study Procedures Method

The acceptability and suitability of the intervention and study procedures were assessed by examining adherence to the study procedures and intervention attendance. Further, participant engagement in the study procedures and intervention was reflected in their capacity and understanding of the intervention. Lastly, acceptability and satisfaction with the intervention were reflected in participants’ motivation and interest in engaging in the gardening activities.

Unique to this program were specific tools that were designed to enhance the acceptability of the intervention. Included was a participant-centered questionnaire, which was used for intervention delivery and planning, that asked if they wanted to work in a group or individually and whether they preferred to work with members of the same or different gender. The questionnaire also asked participants to identify preferred gardening activities and materials from a list provided.

An objective of teacher collaboration was to assess the participants’ capacity and needs related to participation in the gardening tasks beginning in Week 1, including the support required to complete single- and multi-step activities or other learning strategies. Researchers also met with teachers to develop an intervention schedule that accommodated the classroom curriculum. The result was for occupational therapy garden sessions to be delivered in the morning during the scheduled self-care/leisure class sessions.

### 2.7. Intervention and Study Procedures Results

Acceptability was ensured through the development of the following student/participant supports: 1. providing a three-dimensional example to imitate the finished garden product; 2. determining preference for use of paper and pencil vs. tablets to teach garden tasks; 3. applying multi-modal sensory cueing (verbal, visual, tactile, and proprioceptive) to facilitate engagement; 4. providing verbal prompting to support initiation of activities and ongoing cues regarding length of time for activity completion, 5. determining participant preference for working individually or parallel to peers, preferred peers, and group sizes; 6. accommodating sensory preferences such as tactile aversions, preference for low lighting and sound levels; and 7. meeting basic physiological needs before participation, e.g., eating within two hours of school arrival.

Adherence to the intervention was impacted by uncertainty and instability in the participants’ educational and home environment. A minimum of 10 sessions were planned. Participant 1 attended seven sessions due to the need to attend court hearings that reduced school attendance. Participant 2 also had variable attendance, participating in only five sessions due to unexpected and unexplained absences. Participant 3 had to drop out of the study after three sessions due to a disciplinary suspension. It is suggested that length of the program be extended to offset the likelihood of frequent absenteeism. When present, participants were engaged and consistently followed through with task/activity completion. No adverse events were observed or reported.

### 2.8. Preliminary Response to Intervention Method

The intervention protocol was articulated and implemented during this phase of the study. The preliminary participants’ response to intervention was evaluated via visual analysis of pulse rate and HCT during intervention. A VAS was used to measure subjective emotional state changes.

#### 2.8.1. Description of Intervention

This was a 50 min occupational therapy intervention using structured, therapeutic gardening activities. The sessions were led by the research team and took place in the first period of the school day during the ALE’s designated self-care class period (60 min) in the first half of the fall semester. Intervention was introduced after the first week of observations; the program’s frequency varied depending on participant attendance but typically occurred once a week over the following 7-week period. The program had access to a large green space across the street from the campus nearest to a water source. A greenhouse was used to store all gardening materials, tools, and equipment. A shade canopy was also located onsite.

#### 2.8.2. Materials and Setting

At the beginning of the intervention phase, each participant received an unassembled durable wheeled polypropylene gardening cart that they put together. Participants developed group social rules and norms that were posted on a large posterboard and hung on the canopy within participants’ line of sight as a reminder of the expectations while in the garden space. Prior to each session, the researcher set up a 4 × 4 canopy on an outdoor grassy field partially shaded by pine trees. From this location, participants had visual access to the sunrise above the mountains as weather allowed. The workstation shaded by pine trees was adjacent to the canopy and included one table, four chairs, one blanket, and 4” round pillows of assorted firmness and textures. Outdoor temperatures ranged between 68 and 71 degrees throughout the study. The weather ranged from sunny to overcast. The researchers used an easel and a marker to convey written messages and provided visual cues using pictures to simplify each step of the activity.

#### 2.8.3. Sequence of Gardening Sessions

In session 1 and 2, participants collectively assisted with assembly of their “Garden-on-the-Go” mobile stations. For each session thereafter, the participants transported the mobile gardening beds and planting materials to the outside workstation and back to storage. Upon arriving at the workstation, participants chose a quiet place for seating prior to pre-session measures. To begin each session, the researcher provided a visual handout for the session and read the step-by-step instructions out loud, repeating cues as necessary. Throughout the session, soft timed alarms every 10 min oriented the participants to the passage of time. The researcher wrote the session time remaining on a white-board and provided verbal cues to signal 10 min left in the session to facilitate transition from outdoor to indoor. During each session, participants completed gardening activities including assembling mobile raised garden beds; designing the garden beds and planting; planting succulents and seedlings; planting and transplanting seedlings; and propagating houseplants and herbs. At the end of each session, participants assisted with clean-up. The session ended with post-session measures.

### 2.9. Data Analysis Plan

Graphic representations of pulse rate and HCT were employed to assess changes across intervention as well as within each intervention session. Data points reflect scores at the beginning and the end of each treatment session to identify meaningful trends. Additionally, an average pre-intervention and post-intervention pulse rate and interoception score was computed to determine any overall changes that may have been reflected during intervention sessions. An average score was also computed from the VAS pre- and post-intervention to evaluate changes in emotional state.

### 2.10. Preliminary Response to Intervention Results

**Figure 1:**
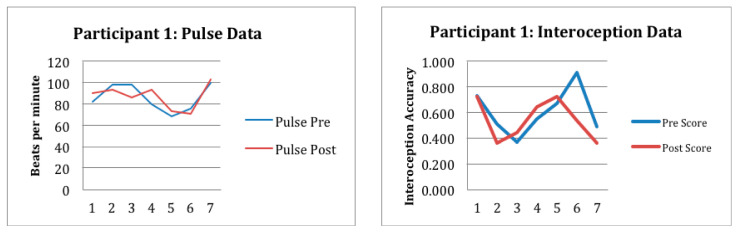
This figure shows the preliminary response to intervention for participant 1 as measured in beats per minute and interoceptive accuracy measurements pre- and post-session.

**Figure 2:**
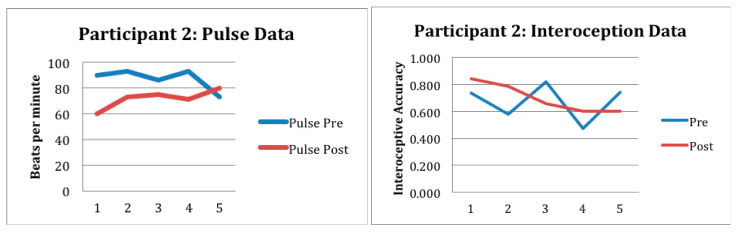
This figure shows the preliminary response to intervention for participant 2 as measured in beats per minute and interoceptive accuracy.

**Figure 3:**
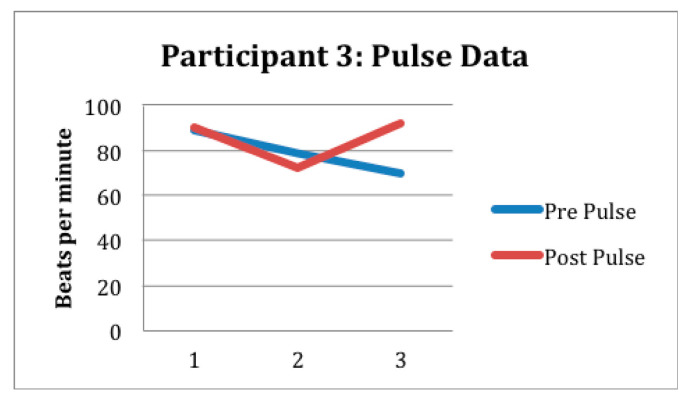
This figure shows the preliminary response to intervention for participant 3 as measured in beats per minute. No interoception accuracy data were available due to absences.

Preliminary outcome data is reported in Figure 1, Figure 2 and Figure 3 and Table 2. As noted previously, participant 1 attended seven sessions and participant 2 attended five sessions. Participant 3 had to drop out of the study after three treatment sessions due to disciplinary suspension. Despite fewer sessions, behavioral observations by the researchers suggested positive participant outcomes of the gardening intervention for participants 1 and 2. Observations included an increase in prosocial behaviors, improved planning, problem solving, turn-taking, as well as better regulation of emotions, expression of empathy, and a sense of meaning and belonging.

Interview feedback from the classroom teacher also suggests that the gardening intervention supported participants’ needs and their academic goals, thus aligning with the role of occupational therapy in educational environments supporting emotional, cognitive, and behavioral outcomes [37]. Examples of teacher feedback for participants 1 and 2 included increased sustained attention, increased motivation to complete class assignments, increased social behaviors, improved relationships with peers, increased positive affect, increased interest in helping peers in academic tasks, and increased interest in community engagement activities related to vocational interests.

Specifically participant 1 and participant 2 had an acceptable level of interoceptive accuracy; the average scores for participant 1 pre- and post-intervention were 0.625 and 0.574; for participant 2, pre- and post-intervention scores were 0.671 and 0.698, respectively. Pulse oximeter data collection was also completed consistently and accurately by the researcher. Although there were no substantial differences noted from the within session data, nor were there any observable trends across sessions, the authors contend that these may still be acceptable measures for this population. See Figure 1, Figure 2 and Figure 3 and Table 2. Variability in interoceptive awareness may exist based on engagement that cannot be reflected in behavioral observation. Future studies are needed to develop a greater understanding of the relationship between interoception, activity engagement, and/or exposure to childhood adversity. It is suggested that interoception measurement be paired with functional measures including observation and evaluation of occupational engagement across contexts.

Similarly, participants did not show much change in emotional state from pre- to post-intervention. Both participants 1 and 2 showed a stable emotional state from pre- to post-intervention with little notable change. See Table 2. It is possible that greater practice in use of this measure might have resulted in more observed change. Additionally, an intervention program lasting longer than five to seven sessions might have produced greater change.

While gardening activities may support interoceptive functions, the HCT may not be the best way to capture the impact of this input for adolescents exposed to adversity. Since there is no universally acceptable level of “accuracy”, the literature suggests caution in interpreting HCT scores solely as a measure of interoceptive ability [34]. Additionally, the literature suggests that adolescents may perform differently from adults due to developmental changes in heart rate perception [38]. Weaker associations were found in adolescents between interoception, emotional awareness, and emotion regulation than previously found in adults. Similarly to this study, multi-method approaches of interoceptive measurement are recommended as well as further refinement of emotion measures [38]. Therefore, we suggest the inclusion of measures of interoceptive sensibility, interoceptive awareness, and/or interoceptive metacognition in future studies [39], such as the Multidimensional Assessment of Interoceptive Awareness- 2 [40], to gain further knowledge as to how gardening can impact interoceptive functions in daily life. It is also hypothesized that accuracy may fluctuate during gardening activities due to the increased attention to other bodily sensations through participation in nature-related activities (e.g., tactile, proprioceptive, smell, and vision).

## 3. Discussion

Preliminarily, the structured, therapeutic gardening program, as a small group activity for adolescents with a history of ACEs and a shared interest in nature, appeared to be an acceptable and suitable therapeutic medium to support overall cognitive, emotional, and behavioral development. The multi-sensory leisure experience inherent within nature environments (i.e., sunlight, water sounds, textures, temperature, and barometric receptors, etc.) presents a potential mechanism for change by targeting complex neural pathways underlying occupational engagement [15,41] in youth with a history of ACEs. The prevalence of uncertainty and instability in the participants’ educational and home environment seemed to influence the feasibility of data collection procedures and sensitivity of outcome measures. These findings underscore the persistent influence of trauma on adolescents with a history of ACEs and point to special considerations for designing trauma-responsive gardening interventions in occupational therapy to support overall health and well-being.

Studies of interoception in clinical psychology suggest that bringing attention to internal bodily sensations may impact emotional processing and identification of emotions [13]. The HCT has been associated with one’s ability to subjectively identify their emotional state, indicating that individuals with higher interoceptive awareness exhibit a greater ability to assess their emotions [4]. An investigation into the relationship between interoception and emotional state has the potential to better inform future intervention needs [31,32]. Such methods would allow for a more robust understanding of the relationship between interoception and engaging in meaningful activities. Studying the impact of gardening interventions on interoceptive accuracy and emotional awareness in this population requires a suitable setting where researchers can account for contextual variation and control for changes in student participation. Lastly, the systemic impact of ACEs necessitates the establishment of extensive collaborative and inclusive initiatives to develop innovative programming to support positive youth development [11,27]. Gardening seemed appropriate for promoting participation in the student role. Shared goals and shared connectedness to nature placed an emphasis on collaboration, intrinsic motivation, and social interaction not readily available in the traditional classroom.

### Limitations

Feasibility study designs have inherent limitations which are also reflected in this study. These include a small sample which restricts generalizability of findings. Additionally, the short duration constrained our ability to assess long-term outcomes. Self-report measures are subject to bias, as are teacher reports and classroom observations. Lastly, absenteeism and the sensitivity of the measures used should be addressed in future studies.

## 4. Lessons Learned

This feasibility study highlights critical considerations for professionals supporting mental health and resilience in adolescents exposed to adversity. Although it is well-accepted that engagement in nature through gardening or other horticultural activities can facilitate a positive impact on health and well-being, the role of occupational therapists and occupational therapy-designed programs is expanding [42]. This feasibility study contributes valuable information regarding the needs of a unique population that can benefit from gardening as a meaningful and health-promoting activity [43].

Firstly, while the setting offered acceptable access to appropriate recruitment, participants, and data collection procedures, infrequent attendance influenced the evaluation of data collection procedures, physiological outcome measures, duration of intervention, and preliminary response to intervention. It is suggested that the duration and frequency of the intervention may not have been optimal and flexible enough for the unpredictability encountered within this population. Previous research suggests that a range of 1 to 4 h spent in gardening is most efficacious [18] and should be considered for future study with this population.

Secondly, a participant-centered, self-reported assessment of gardening interest in our inclusion criteria appears to be an essential component of intervention design. This is corroborated by studies in environmental psychology which identified an interest and attachment to nature as essential to engagement in therapeutic activities as well as a means of developing interoceptive awareness and emotional regulation as protective factors integral to well-being and emotional attachment [15,44,45].

Thirdly, regarding acceptability of outcome measures, more research is needed to capture physiological changes in interoceptive accuracy in adolescents exposed to ACEs in community settings; however, Branham [15]’s structural model of nature connection has identified a predictive relationship between interoceptive awareness (MAIA-2 [40]) and nature [46]. This suggests that the youth version of the MAIA may be an additional self-report measure that captures the functional application of interoceptive abilities in daily life. However, the authors caution to first account for the participants’ literacy level, social desirability, and other characteristics of this measure prior to administration [47].

Lastly, qualitative data appear to be an important reflection of participant outcomes from participation in a nature-based program. Specifically, this study used behavioral ethnographic observations, teacher feedback, and student reports to capture aspects of occupational performance, occupational engagement, and sensory processing. The inclusion of measures that link mental, cognitive, social, behavioral, and emotional domains to participation in daily life is recommended in future studies.

## Figures and Tables

**Table 1:** Demographic characteristics of participants.

Participant	Age	Sex	Criteria 1	Criteria 2	Criteria 3	Criteria 4
P1	16	Male	Child abuse and neglect *	Disruptive behavior	Frequent relocation of residency	ADHD
P2	12	Female	Child abuse and neglect	N/A	History of suicidal ideation/self-harm	Undiagnosed situational anxiety (per school record)
P3	17	Female	N/A	Disruptive behavior	N/A	N/A

Note: * Child abuse and neglect (U.S. Department of Health and Human Services [HHS], 2023).

**Table 2:** Pre–post changes.

	Average Pulse		Avg. Interoceptive Awareness Score		Avg. Emotional State Score	
	Pre	Post	Pre	Post	Pre	Post
P1	86	87	0.625	0.574	3.31	3.44
P2	87	72	0.671	0.698	0	0.25
P3	79	85	n/a	n/a	3.56	2.67

## Data Availability

The original contributions presented in this study are included in the article. Further inquiries can be directed to the corresponding author.
